# Tailoring Properties of Hyaluronate-Based Core–Shell Nanocapsules with Encapsulation of Mixtures of Edible Oils

**DOI:** 10.3390/ijms241914995

**Published:** 2023-10-08

**Authors:** Justyna Bednorz, Krzysztof Smela, Szczepan Zapotoczny

**Affiliations:** 1 Faculty of Chemistry, Jagiellonian University, Gronostajowa 2, 30-387 Krakow, Poland; justyna.bednorz@doctoral.uj.edu.pl; 2 CHDE Polska S.A., Biesiadna 7, 35-304 Rzeszow, Poland; 3 Doctoral School of Exact and Natural Sciences, Jagiellonian University, Prof. St. Lojasiewicza 11, 30-348 Krakow, Poland; 4 Independent Researcher, Chopin St. 7, 35-055 Rzeszow, Poland; krzysztof.smela@gmail.com

**Keywords:** nanocapsules, hyaluronic acid, nanoemulsion, edible oils, interfacial tension

## Abstract

Dispersions of core–shell nanocapsules (nanoemulsion) composed of liquid oil cores and polysaccharide-based shells were fabricated with emulsification using various mixtures of edible oils and amphiphilic hyaluronate derivatized with 12-carbon alkyl chains forming the shells. Such nanocapsules, with typical diameters in the 100–500 nm range, have been previously shown as promising carriers of lipophilic bioactive compounds. Here, the influence of some properties of the oil cores on the size and stability of the capsules were systematically investigated using oil binary mixtures. The results indicated that, in general, the lower the density, viscosity, and interfacial tension (IFT) between the oil and aqueous polymer solution phases, the smaller the size of the capsules. Importantly, an unexpected synergistic reduction of IFT of mixed oils was observed leading to the values below the measured for individual oils. Such a behavior may be used to tailor size but also other properties of the nanocapsules (e.g., stability, solubility of encapsulated compounds) that could not be achieved applying just a single oil. It is in high demand for applications in pharmaceutical or food industries and opens opportunities of using more complex combinations of oils with more components to achieve an even further reduction of IFT leading to even smaller nanocapsules.

## 1. Introduction

Nanocapsules with oil cores and polysaccharide shells have been recently shown as very promising carriers of lipophilic bioactive compounds forming the whole liquid cores or dissolved in plant oils [1,2,3,4]. Aqueous dispersions of such nanocapsules are formed in an emulsification procedure using appropriate amphiphilic derivatives of charged polysaccharides (e.g., hyaluronic acid, chitosan) as a shell-forming polymer stabilizing oil nanodroplets. Unlike nanogels of similar core–shell structure, they do not require crosslinking and no low-molecular weight surfactants are necessary that are crucial for the stabilization of classical nanoemulsions [5]. Aliphatic chains attached covalently to the macromolecules are able to protrude in the oil phase while the main chains of those amphiphilic polysaccharides with exposed ionic groups coat oil droplets ensuring their stabilization in an aqueous medium. The capsules’ size, as shown using cryo-TEM imaging or dynamic light scattering (DLS) measurements, typically varied in the range of 100–500 nm, depending (for example) on the core composition or the length of the aliphatic chains [1]. Importantly, dispersions of such spherical capsules were shown to be stable for at least 15 months, which is crucial for their potential biomedical applications [1].

A remarkable interest in nanoscale delivery systems of bioactive compounds, especially lipophilic ones, is related to their desired functionalities, such as high encapsulation efficiency and loading capacity, and enhanced bioavailability [6]. Encapsulation of lipophilic pharmaceuticals but also nutraceuticals (e.g., carotenoids, curcumin, resveratrol), oil-soluble vitamins, selected lipids, etc. may be beneficial also due to the formation of a protective barrier in such nanocarriers against oxidation, extreme pH, or ionic strength conditions, enhancing the stability of sensitive bioactive compounds and prolonging their biological activity. Encapsulation may also cover unwanted odors and tastes contributing to better sensory properties of encapsulated compounds [7]. Appropriate capsules may modulate the gastrointestinal fate of the encapsulated lipophilic compounds as well as alter their pharmacokinetics and bioavailability [8]. Above all, both in vitro and in vivo studies showed that the oral bioavailability of lipophilic nutraceuticals can be increased substantially upon nanoencapsulation [9].

There is a high potential of applications of nanoemulsions in the food industry, which often relies on additions of hydrophobic functional ingredients including flavors, lipids, preservatives, and vitamins [9,10,11]. Within plant oils, essential oils are also of high interest for the pharma and food industry, but their application may be limited due to, among others, irritation effects on the oesophageal and gastrointestinal mucosa [12]. Thus, nanoencapsulation may be considered as an efficient way to overcome those problems [13].

Nanoscale delivery systems composed of mixtures of vegetable oil containing respective bioactive compounds with nutraceutical properties that have dietary importance may bring particularly beneficial health effects [14], providing that the size, stability, and aggregation behavior of the nanoformulations are not significantly affected by the mixed oil cores. Proper selection of the components of nanoformulations affecting their size and other physicochemical properties is a crucial issue for achieving stability of their aqueous dispersion but also for improving oral delivery and efficient cellular uptake of the nanocarriers with bioactive cargo compounds [15]. In particular, application of mixed oils enables precise tailoring of the properties of the cores in core–shell systems that are important for efficient encapsulation of given active compounds.

The core–shell nanocapsules with hyaluronate-based shells, the subject of this study, were shown to serve as efficient carriers in oral delivery of, e.g., curcumin [4], thanks to their resistance to stomach juice conditions. Additionally, the shell may act as a barrier protecting sensitive cargo molecules (e.g., garlic oil) from oxidation and any destructive environment [3]. A facile fabrication procedure of such nanocapsules allows to easily modify the composition of the cores that can have an impact not only on the loading of the cargo molecules but also on the size and stability of the nanocapsules, which can ultimately affect their bioavailability. However, it is not yet clear how mixing of oils differing in viscosity, density, or polarity will influence the mentioned parameters of the core–shell nanocapsules that are crucial for preserving advantages of nanoformulations [15]. Thus, the main aim of this study is to find structural–functional relationships between the used mixture of oils in the core and the applicability of the created systems for the delivery of various active lipophilic compounds. This systematic study covers nine oils and their binary mixtures that may also be important for the optimization of the composition of other nanoformulations based on lipids (e.g., lipid nanoparticles).

## 2. Results and Discussion

### 2.1. Capsules Composed of Single Oil Cores

**Figure 1:**
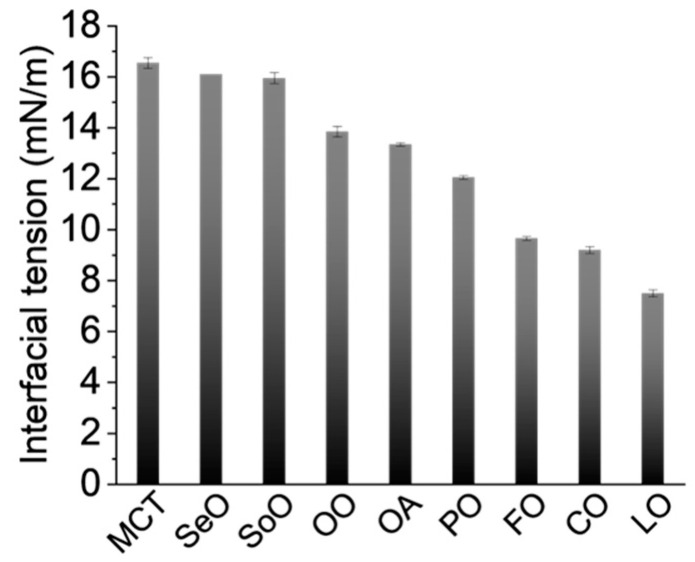
Interfacial tension between an aqueous solution of amphiphilic hyaluronate (HyC12) and different oils together with standard deviations.

**Figure 2:**
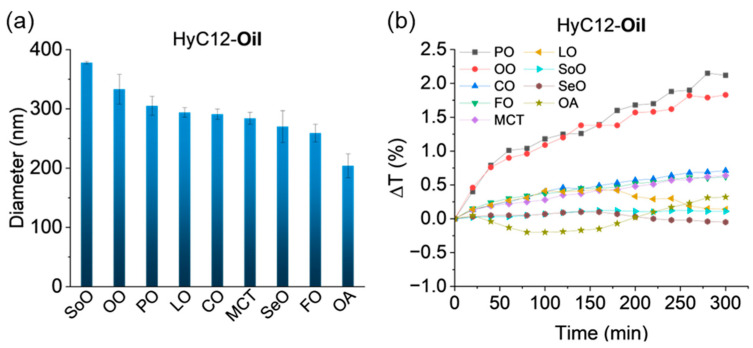
Volume-weighted average hydrodynamic diameters (**a**) and mean variations in transmittance in the lower part of the capsule samples with different oil cores (**b**).

The dispersions of core–shell capsules stabilized with a hyaluronate derivative (HyC12) and varying in the composition of the oil cores were prepared with emulsification and were characterized. A selection of seven plant oils (corn oil (CO), linseed oil (LO), medium-chain triglycerides oil (coconut-based, MCT), olive oil (OO), peanut oil (PO), sesame oil (SeO), soybean oil (SoO)), fish oil (FO), and oleic acid (OA) that differ in density and viscosity were used while the other preparation conditions were kept the same. Table 1 summarizes the values of density, viscosity, and dielectric constant (a measure of solvent polarity) of the tested oils. The IFT between an aqueous solution of the HyC12 and the oils is shown in Figure 1. Figure 2 shows the average hydrodynamic diameters and mean variations in the transmittance in the lower part of the capsule samples with the tested oils. The smallest IFT value of 7.50 mN/m was obtained for LO, while slightly larger IFT values of 9.20 mN/m and 9.65 mN/m were obtained for CO and FO, respectively. For capsules with LO and CO cores, the obtained average hydrodynamic diameters were similar and equal to about 290 nm. When FO was used as a core liquid, diameters were slightly smaller and equal to 260 nm (Figure 2a). Capsules with LO, CO, and FO exhibited similar stability, with transmittance changes not exceeding 0.7% in the studied time (5 h) (Figure 2b). Qualitatively similar trends were observed for the transmittances in the middle and the upper part of the measuring cuvette (Figure S1 in Supplementary Materials).

The IFT values in the range of 12.05–13.95 mN/m were obtained for PO, OA, and OO as the oil phase. Capsules with OA had the smallest average hydrodynamic diameter, equal to 204 nm (it may be underestimated due to the contribution of the self-emulsification of OA [25]. The largest transmittance changes were obtained for the capsules with OO and PO, reaching a maximum of 2.15%. The observed differences between the parameters of the capsules having these oils are most likely due to variations in the oil densities and viscosities. OA had a lower density and viscosity than OO and PO. Capsules with PO, OO, and SoO had the largest average diameters (305–378 nm). These oils also had relatively high viscosities, and their densities were similar. The largest IFT values were obtained for SoO, SeO, and MCT (15.95–16.55 mN/m). The average capsule sizes of SeO and MCT cores were found to be similar: 270 nm and 284 nm, respectively. The volume-weighted distributions of diameters of the capsules with different oils are shown in Figure S2 (Supplementary Materials).

After analysis, it is not possible to claim that there is a single dominating parameter of the core liquid or an IFT value that can govern the size and stability of the obtained capsules. Dielectric constants of the applied oils vary from 2.377 for OA to 3.930 for MCT; however, the overall variations are not large (Table 1) and also dependent on, e.g., frequency of the electric field applied and temperature. Thus, it would be unreliable to conclude the effect of the dielectric constant of the oil phase on the parameters of the capsules. While the studied oils (except for OA) are complex mixtures of various glycerides, proteins, sterols, free fatty acids, etc., the bulk properties of the liquids and also their minor components affecting the IFT may play an important role in the formation of nanoemulsions [26]. Nevertheless, both lower viscosity and lower IFT values seem to generally favor the formation of smaller capsules and higher dispersion stability, which are commonly desired. The correlation of density of the core (within the studied range: 0.89–0.946 g/cm^3^) or its polarity was not clear in the studied systems. However, using the mixture of oils, some parameters may be varied systematically while keeping the other parameters practically unchanged, and some parameters of the capsules may be tailored for desired applications using this method.

### 2.2. Capsules Containing Binary Mixtures of Oils—Effects of Density

The correlations between the oil phase density and the capsules’ size and stability of the emulsion were studied via encapsulating the mixtures of OA and MCT, since MCT has a much higher density (0.950 g/cm^3^) than OA (0.899 g/cm^3^), while their viscosities were similar (Table 1) and the IFT values differed by less than 20% (Figure 1).

**Figure 3:**
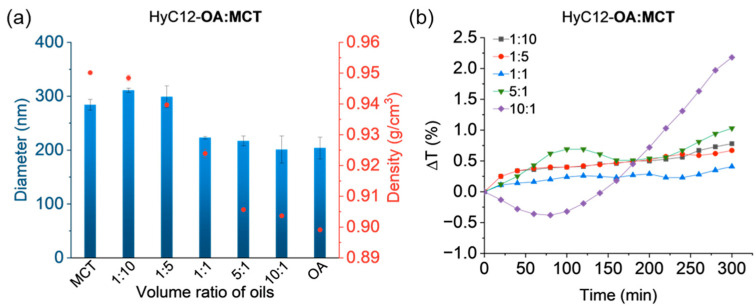
Volume-weighted hydrodynamic diameters (blue bars) of capsules and densities (red dots) of the respective oil mixtures serving as the cores (**a**); mean variations in transmittance in the lower part of the capsules dispersions (**b**). Hydrodynamic diameters and density data are presented as mean ± standard deviation (SD); for hydrodynamic diameters, n = 3; for densities, n = 2. (Detailed statistical analysis of the data is presented in Figure S4).

PDI values for the dispersions of capsules with cores made of mixtures of oils of different densities were relatively small and did not exceed 0.347 (Table S1 in Supplementary Materials). The hydrodynamic diameters of the capsules were found to decrease with the increasing content of OA, which can be correlated with the decrease of the core density (Figure 3a and Figure S3 in Supplementary Materials). The density of the oil mixtures ranged from 0.904 g/cm^3^ and a volume ratio of 10:1 to 0.948 g/cm^3^ and a volume ratio of 1:10. Diameters of about 300 nm were obtained for capsules with OA:MCT volume ratios of 1:10 and 1:5, while the sizes of capsules with the oil ratios of 1:1, 5:1, and 10:1 were about 200 nm. Some instabilities in the set of samples could be observed for the capsules with the lowest oil phase density (Figure 3b). For those capsules (10:1, OA:MCT), the transmittance decreased for the first 80 min, followed by an increase; however, the maximum change did not exceed 2.2%. A sample of HyC12-MCT:OA with a volume ratio of oils equal to 5:1 exhibited even lower variations of transmittance—the changes were seen in the lower part of the sample, while in the middle and upper part of the sample the emulsion seemed to be more stable (Figure S5 in Supplementary Materials). In the lower part of the sample, the transmittance initially slightly increased, followed by a decrease and then an increase again; however, the changes amounted to a maximum of 1%. Mean variations in transmittance for the other samples did not exceed 0.8%; hence, the stability of the capsules seemed to be slightly higher when a higher-density oil phase was used. It might be explained by buoyant forces that affect capsules that have a density different than water. Some instabilities were also observed for the capsules based only on low-density OA liquid, especially following turbidimetric measurements in the middle and upper parts of the cuvette (Figure S1 in Supplementary Materials). Nevertheless, the measured variations of the transmittance in time were relatively small for all the studied systems, indicating their sufficiently high stability.

### 2.3. Effect of Viscosity

To study the correlation of the oil phase viscosity and the capsules’ parameters, mixtures of OA:OO and MCT:SoO were encapsulated. The oils were selected to differ in viscosity but had a similar density and IFT between the aqueous and oil phases (Table 1 and Figure 1).

**Figure 4:**
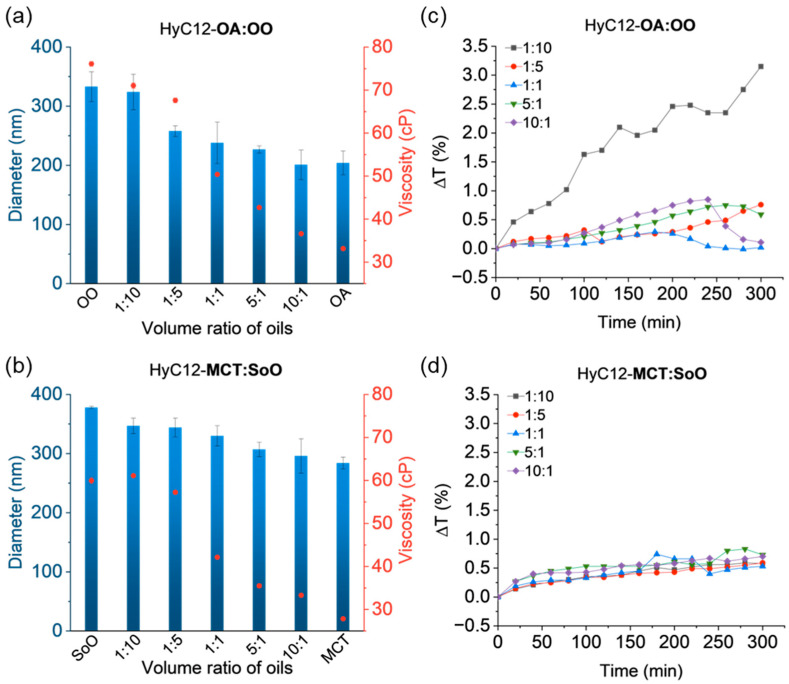
Volume-weighted hydrodynamic diameters (blue bars) of the capsules and respective viscosities (red dots) of the oil mixtures (**a**,**b**); mean variations in transmittance in the lower part of the capsules’ dispersions (**c**,**d**). Hydrodynamic diameters and viscosity data are presented as mean ± standard deviation (SD); for hydrodynamic diameters, n = 3; for viscosities, n = 2. (Detailed statistical analysis of the data is presented in Figure S4).

For mixtures of OA:OO, the viscosity values varied in the range of 36.6–71.1 cP, while for mixtures of MCT:SoO the values were in the range of 33.3–61.1 cP. After analysing the sizes obtained for the capsules with these oil mixtures, a decrease in size was observed with the increasing content of the low viscosity oil. For capsules with OA:OO with a volume ratio of the oils equal to 1:10, the average size was about 320 nm, while for capsules with the ratio of 10:1, the diameters were found to be smaller than 200 nm (Figure 4a). For HyC12-MCT:SoO, the trend was not so much pronounced; however, the sizes varied in the range of ca. 350 nm to below 300 nm while decreasing the viscosity of the oil phase (Figure 4b). No clear effect of viscosity on the capsules’ stability was observed. For the capsules with mixtures of OA:OO, the largest changes in transmittance were obtained for a volume ratio of oils of 1:10 (Figure 4c and Figure S6 in Supplementary Materials). The transmittance increased non-uniformly but did not exceed 3.15%. For other samples, the changes were much smaller, with a maximum of 0.85%. In the case of HyC12-MCT:SoO, very low transmittance changes, not exceeding 0.74%, were obtained for all volume ratios of the oils, implying enhanced stability of such systems. PDI values for the capsule with mixtures of OA:OO oils were relatively small and varied in the range of 0.174–0.332, while for MCT:SoO mixtures, the values were in the range of 0.182–0.228 (Table S1).

### 2.4. Effect of Interfacial Tension

**Figure 5:**
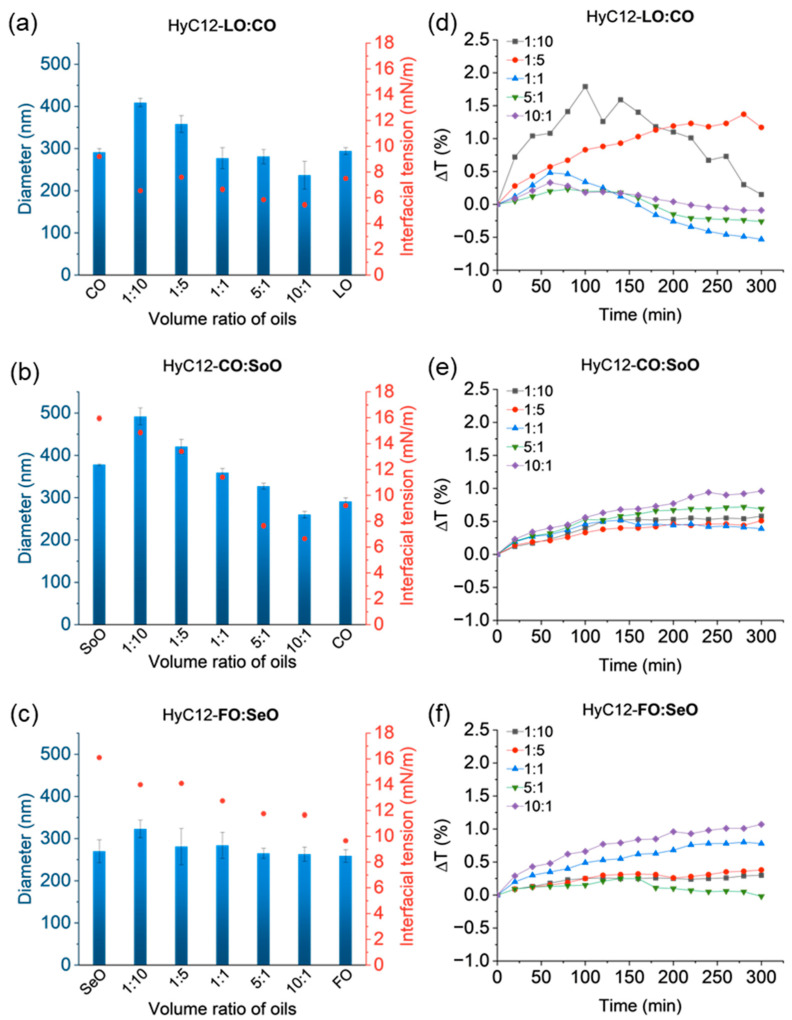
Volume-weighted hydrodynamic diameters (blue bars) of the capsules and respective IFT values (red dots) of the oil mixtures (**a**–**c**); mean variations in transmittance in the lower part of the capsules dispersions (**d**–**f**). Hydrodynamic diameters and IFT data presented as mean ± standard deviation (SD); for hydrodynamic diameters, n = 3; for interfacial tension values, n = 2. (Detailed statistical analysis of the data is presented in Figure S4).

The effect of IFT was studied by encapsulating mixtures of LO:CO, CO:SoO, and FO:SeO oils. The oil pairs were selected to have similar density and viscosity but different IFT values versus the HyC12 polymer solution (Figure 1). The hydrodynamic diameters, the respective IFT values for the oil mixtures, and the mean variations in transmittance in the lower part of the capsule samples with oil mixtures with different IFT values are summarized in Figure 5. The obtained PDI values for those capsules were relatively small and varied in the range of 0.187–0.329 (Table S1). For LO:CO, the pair IFT values did not differ substantially (LO—7.5 mN/m and CO—9.2 mN/m, Figure 1). Despite this, the values of IFT between the aqueous phase and LO:CO oil mixtures with different volume ratios indicate that the values decrease with increasing LO content reaching ca. 5.5 mN/m for 5:1 and 10:1 ratios (Figure 5a) and are significantly below the surface tension measured for pure LO. The variations of IFT at w/o the interface for binary oil mixtures can be explained by compositional changes at the interface; however, no such effects were observed in typical binary mixtures [27]. Moreover, even when considering the complex compositions of each applied edible oil, such a reduction of IFT (below the value for each pure oil) seems to also not be predictable by more recent simulations even for multicomponent mixtures considering only preferential interfacial accumulation of polar oil components [28]. Thus, another mechanism of such synergistic reduction of the IFT should be considered that may involve, e.g., conformational changes of the polymer at the interface (detailed elaboration of the mechanism is beyond the scope of this report). Nevertheless, this unexpected synergistic effect may be utilized in tailoring the properties of the nanocapsules that cannot be achieved by varying just pure oils alone.

For CO and SoO, the difference in IFT is even larger than for the LO and CO pair (SoO—16.0 mN/m; CO—9.2 mN/m, Figure 1). Similarly, for a CO:SoO volume ratio of 10:1, an IFT as low as 6.7 mN/m (Figure 5b) was achieved, which is much lower than the value observed for pure CO. Moreover, the largest variations of IFT values were obtained for this oils pair (6.7–16.0 mN/m). There is a similar difference in IFT between the FO and SeO oils and the SoO and CO oils, which equal to about 7 mN/m. However, the differences in IFT between the aqueous phase and FO:SeO oil mixtures of individual volume ratios are much smaller—the values oscillate between 11.7 and 14.1 mN/m (Figure 5c). The capsules’ sizes obtained for the different volume ratios of the oils indicate that the smaller IFT is between the aqueous and oil phase, the smaller the hydrodynamic diameters of the capsules are, which was not unequivocal when encapsulating the oils alone (compare Figure 1 and Figure 2). For HyC12-LO:CO, the hydrodynamic diameters ranged from 410 nm for an oil phase with a volume ratio of 1:10 down to 240 nm for the ratio of 10:1 (Figure 5a). The diameters of HyC12-CO:SoO mixtures ranged from 490 nm for volume ratio of the oils equal to 1:10 down to 260 nm for the ratio of 10:1 (Figure 5b). For HyC12-FO:SeO, the size differences were not as large as for HyC12-LO:CO and HyC12-SoO:CO. The diameters of HyC12-FO:SeO ranged from 320 nm for a volume ratio of oils equal to 1:10 down to 260 nm the ratio of 10:1 (Figure 5c). The reason for such small size differences for HyC12-FO:SeO with various oil ratios may be due to small variations in the respective IFT values (Figure 5c).

When using an oil phase consisting of LO:CO mixtures, smaller transmittance changes were observed for the oil phase capsules with a lower IFT, with volume ratios of 5:1 and 10:1 (Figure 5d and Figure S7) indicating higher stability of such systems. For the capsules with an oil volume ratio of 1:10, the transmittance increased and then decreased during the experiment; however, the overall changes did not exceed 1.8%. For the capsule samples with CO:SeO and FO:SeO mixtures, the transmittance changes were similar for all the studied samples within a given set (somehow larger for low IFT systems) (Figure 5e,f), indicating a rather weak influence of IFT on the stability of the studied systems.

### 2.5. Mixtures of Oils with Similar Bulk Properties

**Figure 6:**
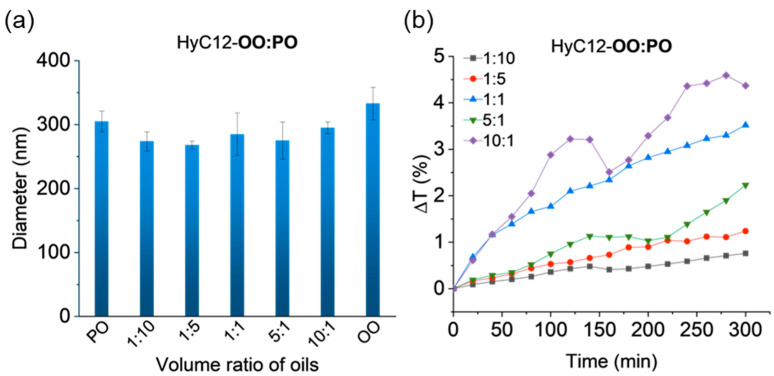
Volume-weighted hydrodynamic diameters (**a**) and mean variations in transmittance in the lower part of the capsule samples with a mix of oils with similar physical properties (**b**). Hydrodynamic diameters data are presented as mean ± standard deviation (SD); n = 3. (Detailed statistical analysis of the data is presented in Figure S4).

Mixtures of oils with similar bulk physicochemical properties were also encapsulated—the OO and PO pair was selected for this purpose. Hydrodynamic diameters of the capsules with mixtures of these oils with different volume ratios are similar and oscillate around values of 274–295 nm (Figure 6a). This is consistent with insignificant variations in the density, viscosity, and IFT of the oils. The largest transmittance changes of up to 4.6% were obtained for capsules with oil mixtures with a volume ratio of 10:1, while the smallest transmittance changes were obtained for capsules with an oil volume ratio of 1:10 (Figure 6b and Figure S8). The results can be rationalized by considering that both oils produced the least stable capsules (within the oils studied here) when encapsulated alone.

## 3. Materials and Methods

Hyaluronic acid sodium salt from *Streptococcus equi* (Mw ≈ 150,000 g/mol); dodecylamine (>99.5%, GC); 1-ethyl-3-(3-dimethylaminopropyl) carbodiimide hydrochloride (EDC, >99.0%); N-hydroxysuccinimide (NHS, 98%); tert-butanol (99.0%); oleic acid (OA; PhEur); corn oil (CO); fish oil from menhaden (FO); soybean oil (SoO); sesame oil (SeO); peanut oil (PO); and linseed oil (LO) were purchased from Sigma-Aldrich (Poznan, Poland). Medium-chain triglycerides oil (coconut based, MCT) and olive oil (OO) were purchased from Gustav Heess (Warsaw, Poland). Sodium chloride (NaCl, 99.5%) was purchased from Avantor Performance Materials Poland S.A. (Gliwice, Poland). Chloroform (99.8%) was purchased from Chempur (Piekary Slaskie, Poland). Dimethylformamide (DMF, p.a.) was purchased from Lachner (Neratovice, Czech Republic). Deionized water was used in all the experiments.

### 3.1. Hydrophobic Modification of Hyaluronate

The synthesis of hyaluronate modified with a hydrophobic 12-carbon alkyl chain (HyC12) [1]. Briefly, sodium hyaluronate was dissolved in water (5 g/L) and heated to 37 °C. Once the set temperature was reached, 45 mg of EDC, 27 mg of NHS, and 10 mL of water were added. The mixture was stirred for 30 min; then, 80 mL of DMF, 20 mL of chloroform, and 2 mL of 0.1 M solution of dodecylamine in chloroform were added. The mixture was stirred on a magnetic stirrer for 24 h at 37 °C. In the first purification step, the product was dialyzed into a mixture of a 1-fold diluted phosphate buffer (PBS, c = 0.1 M, pH = 7.4) and tert-butanol (1:1, *v*/*v*) and was then dialyzed into distilled water. The purified product was finally freeze-dried.

### 3.2. Capsule Preparation

The procedure of capsule preparation was carried out according to a previously described procedure [1]. Briefly, the corresponding oil or mixtures of oils were added to a polymer solution (HyC12) (1 g/L in 0.15 M NaCl) in a volume ratio of 1:200. The mixtures were shaken with a vortex shaker (IKA, Königswinter, Germany) for 15 min and were then sonicated for 30 min in an ultrasonic bath (540 W, Sonic-6, Polsonic, Warsaw, Poland). Mixtures of oils with volume ratios of 1:10, 1:5, 1:1, 5:1, and 10:1 (*v*/*v*) were used for the tests.

### 3.3. Density Measurements

The density of the mixtures of oils with different volume ratios was determined by weighing a fixed volume of the corresponding mixture.

### 3.4. Viscosity Measurements

The viscosity of the mixtures of oils was measured at a shear rate of 25 s^−1^ using a First Plus rotational viscometer (Lamy Rheology, Champagne-au-Mont-d’Or, France).

### 3.5. Measurements of Interfacial Tension

The interfacial tension (IFT) between the aqueous solution of the HyC12 and the corresponding oil phase was measured at ambient temperature using a Tensiometer K9 tensiometer (Kruss, Hamburg, Germany) with the ring method. The ring was immersed in the aqueous solution of the polymer (HyC12, 1 g/L in 0.15 M NaCl), and then the oil phase was introduced. After 15 min of equilibrium establishment, the ring was slowly pulled out and the IFT value was measured. Each experiment was repeated, and the data were averaged.

### 3.6. Turbidimetry

Stability measurements of the capsules’ dispersions were carried out using a Turbiscan optical analyzer (TurbiSoft Classic 2.2.0.101, Formulaction, Toulouse, France). A dispersion of nanocapsules was placed in a glass cuvette and the data showing changes in the solution transmittance in the lower (0–3 mm from the bottom of cuvettes), middle (10–20 mm), and upper part of the sample (35–40 mm) were collected. Signals were recorded every 20 min for 5 h.

### 3.7. Dynamic Light Scattering

Hydrodynamic diameters of the nanocapsules were measured using the DLS technique at 25 °C with a Zetasizer Nano Series instrument (Malvern Instruments, Malvern, UK) with a detection angle of 173°. For measurements, native samples were diluted 100 times. The general-purpose mode was used. The data represent the average result from 3 measurements, each consisting of 6 runs, along with the standard deviation.

## 4. Conclusions

Polymer capsules containing liquid oil cores and hyaluronate-based shells were fabricated with emulsification using various edible oils and, particularly, their mixtures to study the influence of the selected oil parameters on the size and stability of the capsules. Nine oils, mostly plant oils, but also a fish oil and pure oleic acid differing in density, viscosity, and the interfacial tension (IFT) against the aqueous solution of the shell-forming derivative of hyaluronate (HyC12) were selected. Systematic variation of only a given parameter was made possible thanks to application of seven binary mixtures of oils that differed significantly within a pair in a given parameter—only while keeping the other parameters similar. In general, the results indicated the same trends for all three individual parameters—the lower the density, viscosity, or IFT of the studied mixtures, the smaller the hydrodynamic diameters of the capsules that varied in the range of ca. 200–500 nm. There was no clear trend observed for PDI that was not exceeding 0.35 (typically around 0.2), indicating that there was a relatively narrow size distribution of the capsules, disregarding the properties of the oil cores. These are important findings for tailoring the nanocapsules’ diameters for applications aiming at specific sizes of such carriers as well as the solubility of given actives (lipophilic pharmaceutical, nutraceuticals, etc.) in the oil cores for optimizing their bioavailability. Stability of the formed nanoemulsions was also followed using turbidimetric measurements; however, no clear trends could be observed as all the studied systems were relatively stable within the studied period.

Importantly, an unexpected synergistic reduction of IFT in some pairs of mixed oils was observed, leading to values of individual oils being and as low as 5.5 mN/m (component oils: linseed oil—7.5 mN/m; corn oil—9.2 mN/m). This can be explained in terms of mutual interactions, which lead to a higher occupation of the interface of some complementary components (even minor) of the mixed individual oils that are in fact multicomponent mixtures of various compounds (except for OA). Due to the complexity of such mixtures, it would be difficult to predict the actual occurrence of the synergistic reduction of IFT for a given pair of oils, and clarifying such an effect requires further systematic studies. Nevertheless, such a behavior may be used to tailor the size and further enhance long-term stability of the nanocapsules and is unlikely to be achieved by applying a single edible oil. Such results are crucial for further applications of the oil-core hyaluronate-based shell nanocapsules in pharmaceutical or food industries, opening opportunities of using more complex combinations of oils with more components to achieve even smaller (below 100 nm) nanocapsules that are in demand due to, e.g., enhanced bioavailability.

## Figures and Tables

**Table 1:** Physicochemical parameters: density (d), viscosity (η), and dielectric constant (ε) of the studied oils at 25 °C.

Oil	d (g/cm^3^)	η (cP)	ε	References
Corn Oil (CO)	0.916	52.3 ^a^	3.127	d: [16], η: [17], ε: [18]
Fish Oil (FO)	0.93	50	-	d: safety data sheet, Sigma-Aldrich, η: [19]
Linseed Oil (LO)	0.93	53 ^b^	3.87 ^c^	d: safety data sheet, Sigma-Aldrich, η: [20], ε: [21]
Medium-Chain Triglycerides (MCT)	0.946 (0.950) ^f^	25.5 (27.9) ^g^	3.930 ^d^	d: [22], η: [22], ε: [23]
Oleic Acid (OA)	0.89 (0.899) ^f^	29 ^a^ (33.2) ^g^	2.377	d: safety data sheet, Sigma-Aldrich, η: [17], ε: [18]
Olive Oil (OO)	0.915	56.2 ^e^ (76.1) ^g^	3.062	d: safety data sheet, Sigma-Aldrich, η: [24], ε: [18]
Peanut Oil (PO)	0.91	57.4 ^e^	3.62 ^c^	d: safety data sheet, Sigma-Aldrich, η: [24], ε: [21]
Sesame Oil (SeO)	0.92	52.5 ^e^	3.110	d: safety data sheet, Sigma-Aldrich, η: [24], ε: [18]
Soybean Oil (SoO)	0.917	54.3 ^a^ (60.0) ^g^	3.115	d: safety data sheet, Sigma-Aldrich, η: [17], ε: [18]

Experimental data measured at ^a^ 23.9 °C; ^b^ 20 °C; ^c^ 40 °C; ^d^ 30 °C; ^e^ 26 °C; ^f^ measured here at 21 °C; ^g^ measured here at 24 °C.

## Data Availability

Data are contained within this article and the Supplementary Materials.

## Supplementary Materials

The following supporting information can be downloaded at: https://www.mdpi.com/article/10.3390/ijms241914995/s1.
