# Supplementary material for: Tailoring Properties of Hyaluronate-Based Core–Shell Nanocapsules with Encapsulation of Mixtures of Edible Oils
Source: Int J Mol Sci. 2023 Oct 8;24(19):14995. doi: 10.3390/ijms241914995 (PMC10573177; doi:10.3390/ijms241914995)
Supplement: Supplementary file 1 [file ijms-24-14995-s001.zip › ijms-2604569-supplementary.pdf]

Supplementary material

Tailoring properties of hyaluronate-based core-shell nanocapsules by encapsulation of mixtures of edible oils

Justyna Bednorz [1,2,3], Krzysztof Smela [4] and Szczepan Zapotoczny [1,*]

1 Jagiellonian University, Faculty of Chemistry, Gronostajowa 2, 30-387 Krakow, Poland; justyna.bednorz@doctoral.uj.edu.pl (J.B.), s.zapotoczny@uj.edu.pl (S.Z.)
2 CHDE Polska S.A., Biesiadna 7, 35-304 Rzeszow, Poland
3 Jagiellonian University, Doctoral School of Exact and Natural Sciences, Prof. St. Lojasiewicza 11, 30-348 Krakow, Poland
4 Independent researcher, Chopin St. 7, 35-055 Rzeszow, Poland; krzysztof.smela@gmail.com (K.S.)
* Correspondence: s.zapotoczny@uj.edu.pl

Table S1. Polydispersity index values from DLS measurements of various dispersions of the capsules.

**Sample**	**Polydispersity index (PDI)**
**Capsules composed of single oil cores**	
HyC12-CO	0.327
HyC12-FO	0.291
HyC12-LO	0,259
HyC12-MCT	0.194
HyC12-OA	0.206
HyC12-OO	0.249
HyC12-PO	0.220
HyC12-SeO	0.326
HyC12-SoO	0.186
**Capsules containing binary mixtures of oils – effects of density**	
HyC12-OA:MCT 1:10	0.216
HyC12-OA:MCT 1:5	0.250
HyC12-OA:MCT 1:1	0.313
HyC12-OA:MCT 5:1	0.347
HyC12-OA:MCT 10:1	0.231
**Capsules containing binary mixtures of oils – effects of viscosity**	
HyC12-OA:OO 1:10	0.263
HyC12-OA:OO 1:5	0.184
HyC12-OA:OO 1:1	0.332
HyC12-OA:OO 5:1	0.174
HyC12-OA:OO 10:1	0.222
HyC12-MCT:SoO 1:10	0.228
HyC12-MCT:SoO 1:5	0.182
HyC12-MCT:SoO 1:1	0.190
HyC12-MCT:SoO 5:1	0.205
HyC12-MCT:SoO 10:1	0.215
**Capsules containing binary mixtures of oils – effects of interfacial tension**	
HyC12-LO:CO 1:10	0.234
HyC12-LO:CO 1:5	0.194
HyC12-LO:CO 1:1	0.301
HyC12-LO:CO 5:1	0.260
HyC12-LO:CO 10:1	0.329
HyC12-CO:SoO 1:10	0.278
HyC12-CO:SoO 1:5	0.187
HyC12-CO:SoO 1:1	0.253
HyC12-CO:SoO 5:1	0.262

HyC12-CO:SoO 10:1	0.221
HyC12-FO:SeO 1:10	0.245
HyC12-FO:SeO 1:5	0.254
HyC12-FO:SeO 1:1	0.221
HyC12-FO:SeO 5:1	0.212
HyC12-FO:SeO 10:1	0.316
**Capsules containing mixtures of oils with similar bulk properties**	
HyC12-OO:PO 1:10	0.192
HyC12-OO:PO 1:5	0.201
HyC12-OO:PO 1:1	0.228
HyC12-OO:PO 5:1	0.212
HyC12-OO:PO 10:1	0.194

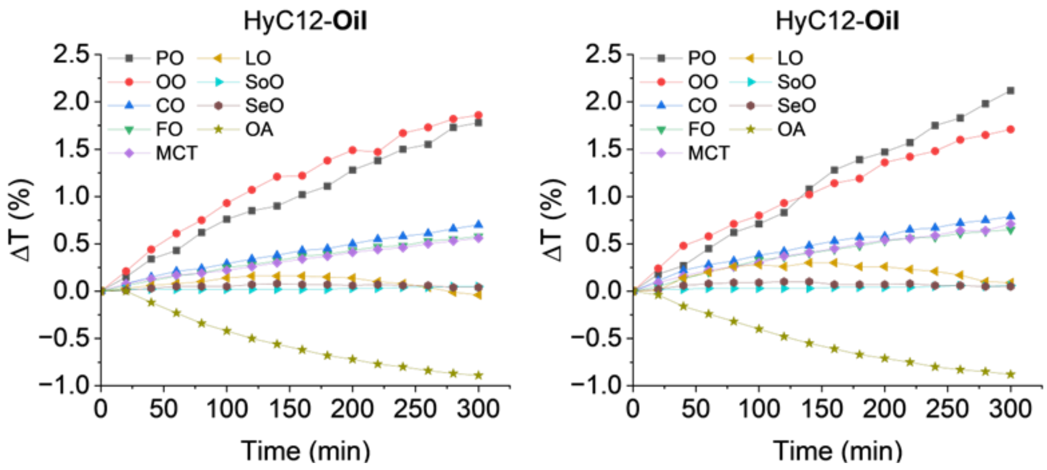

Figure S1. Mean variations in transmittance in the middle (**a**) and upper (**b**) parts of the capsule samples with different oils.

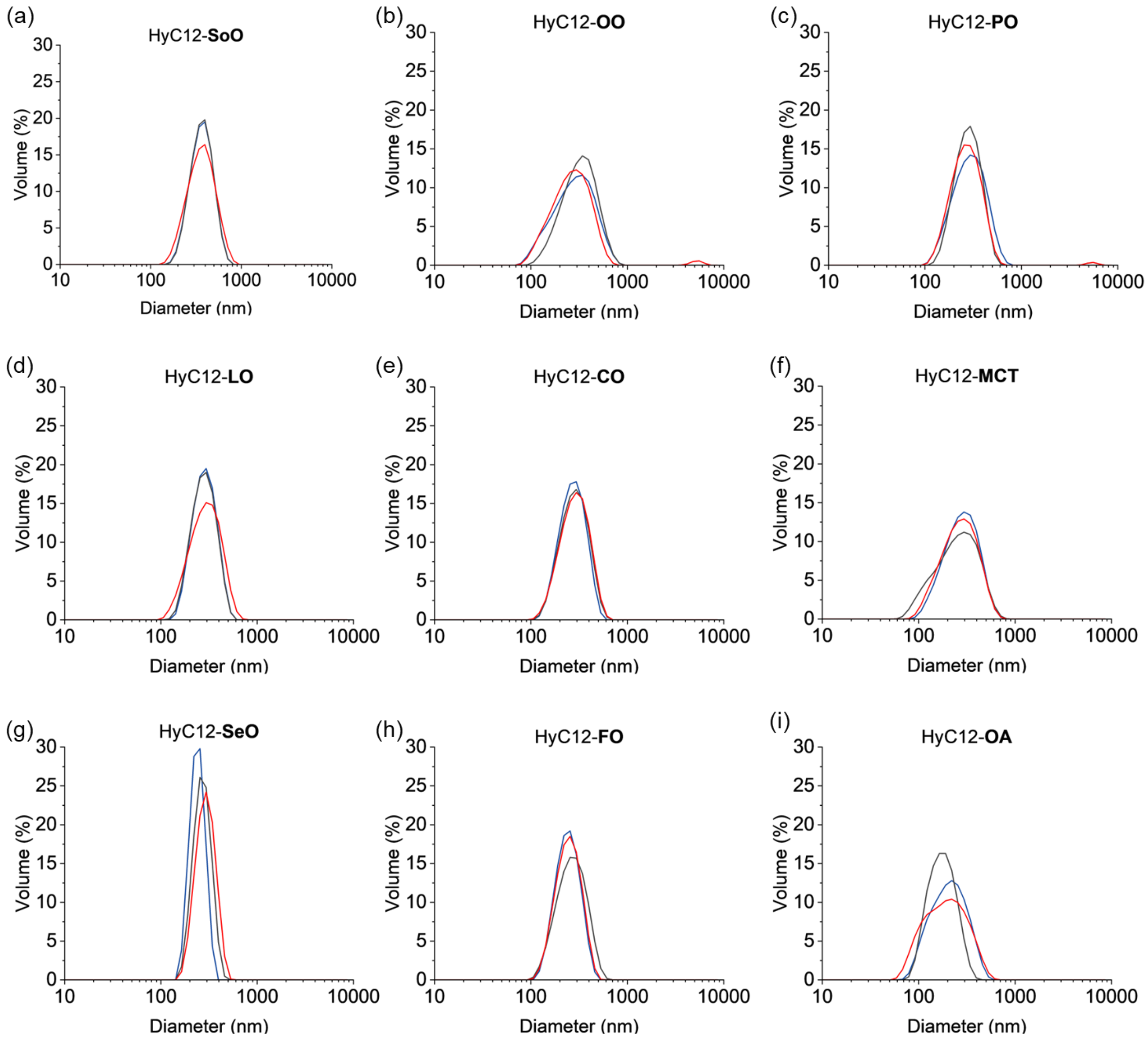

Figure S2. Volume-weighted distributions of diameters of the capsules with different oil core: (a) SoO, (b) OO, (c) PO, (d) LO, (e) CO, (f) MCT, (g) SeO, (h) FO, (i) OA.

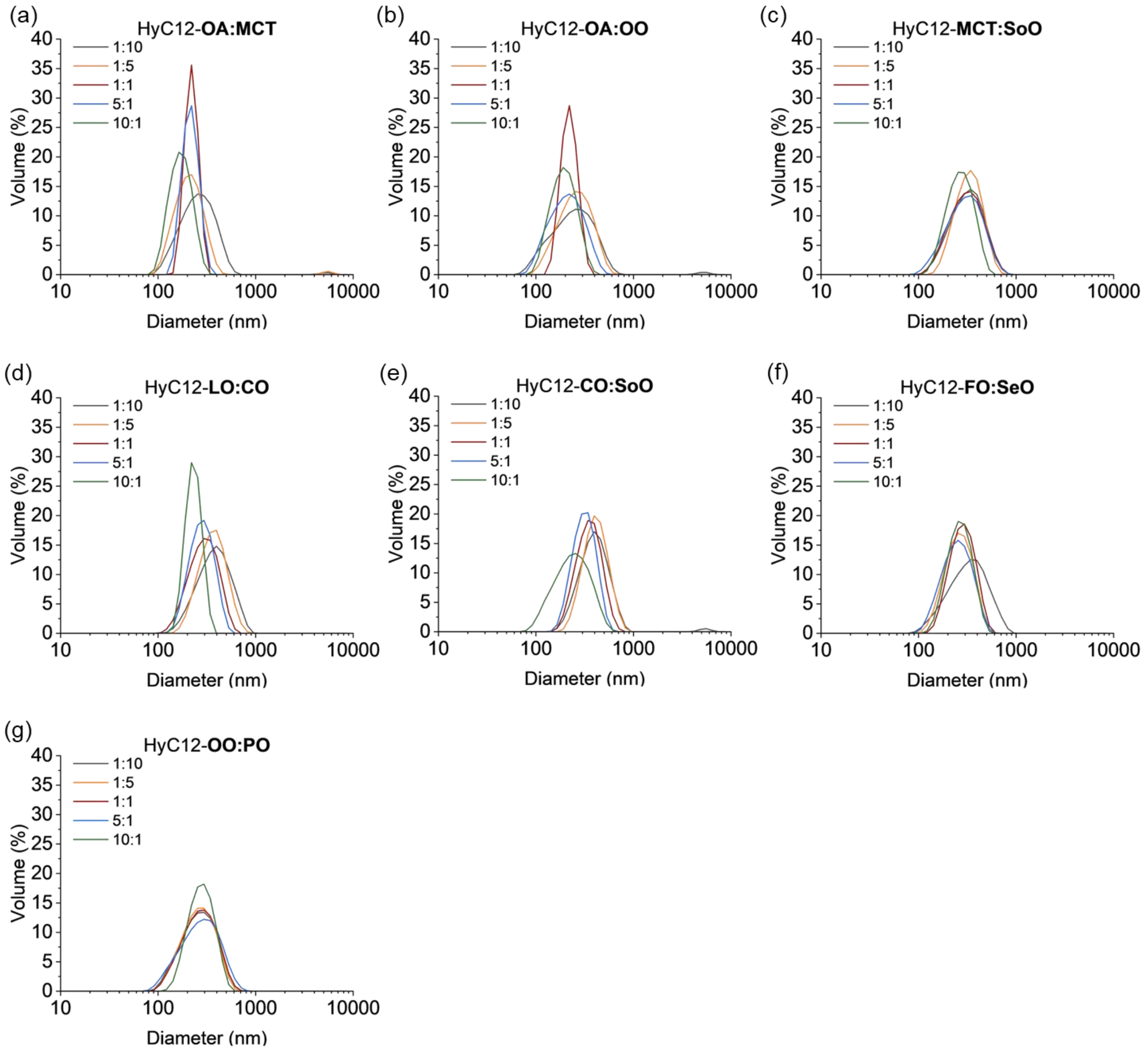

Figure S3. Volume-weighted distibutions of diameters of the capsules with the cores composed of mixtures of oils: (a) OA and MCT, (b) OA and OO, (c) MCT and SoO, (d) LO and CO, (e) CO and SoO, (f) FO and SeO, (g) OO and PO.

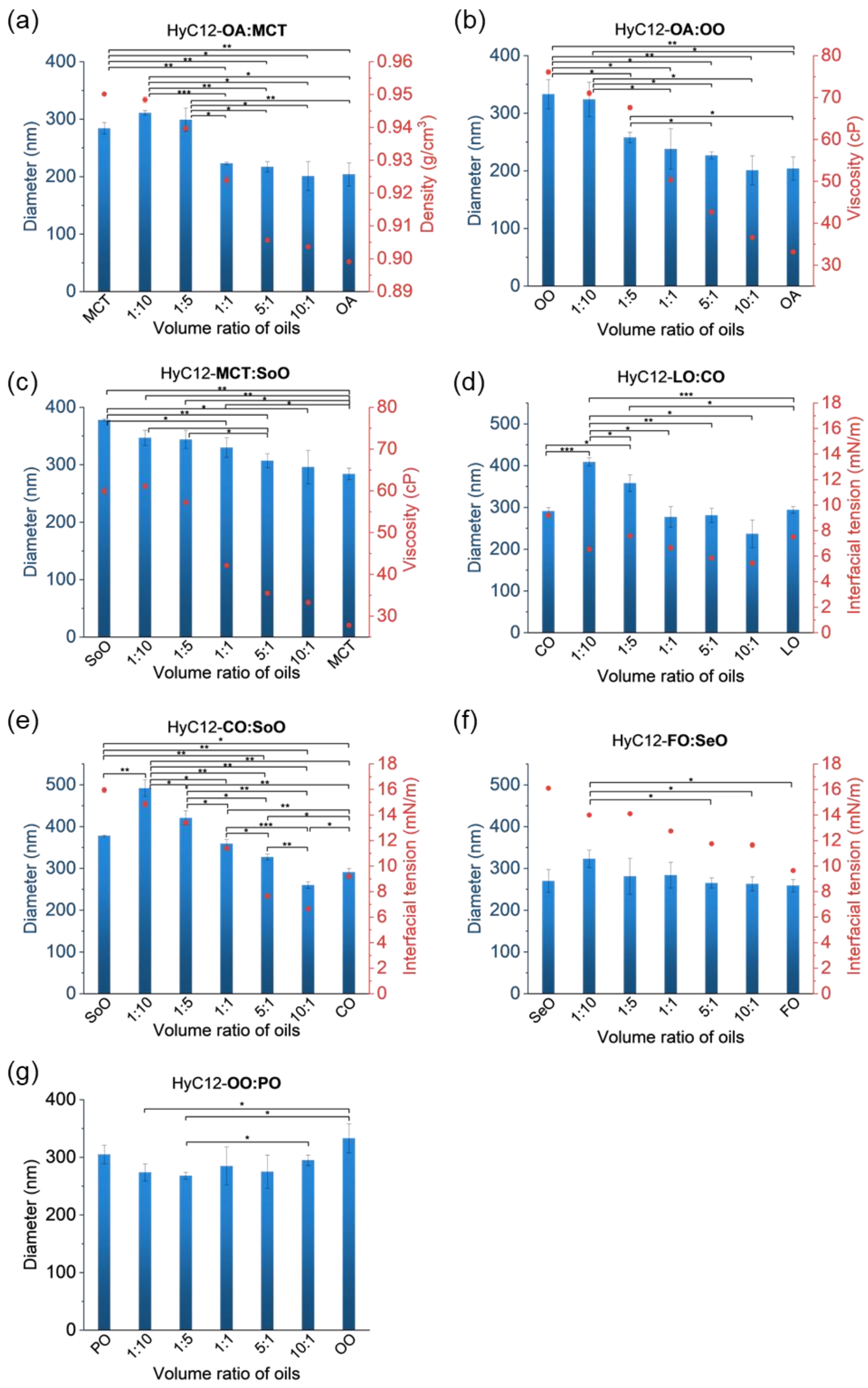

Figure S4. Volume-weighted hydrodynamic diameters (blue bars) of the capsules with the cores composed of various mixtures of oils: (a) OA and MCT, (b) OA and OO, (c) MCT and SoO, (d) LO and CO, (e) CO and SoO, (f) FO and SeO, (g) OO and PO shown together with respective properties of the oil cores (density, viscosity or IFT; as red dots) presented as mean +/- standard deviation (SD); for hydrodynamic diameters n = 3; for density/viscosity/interfacial tension values n = 2. Statistical significance between samples of the capsules with different volume ratios of oils was estimated using a two-tailed Student's t-test; * $p < 0.05$; ** $p < 0.01$; *** $p < 0.001$.

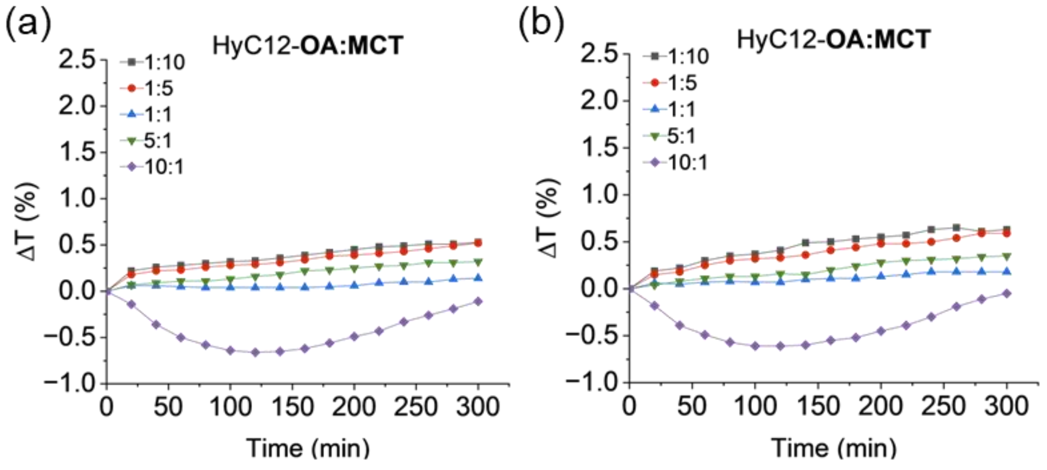

Figure S5. Mean variations in transmittance in the middle (**a**) and upper (**b**) parts of the capsule samples with mixtures of oils (OA and MCT) of different densities.

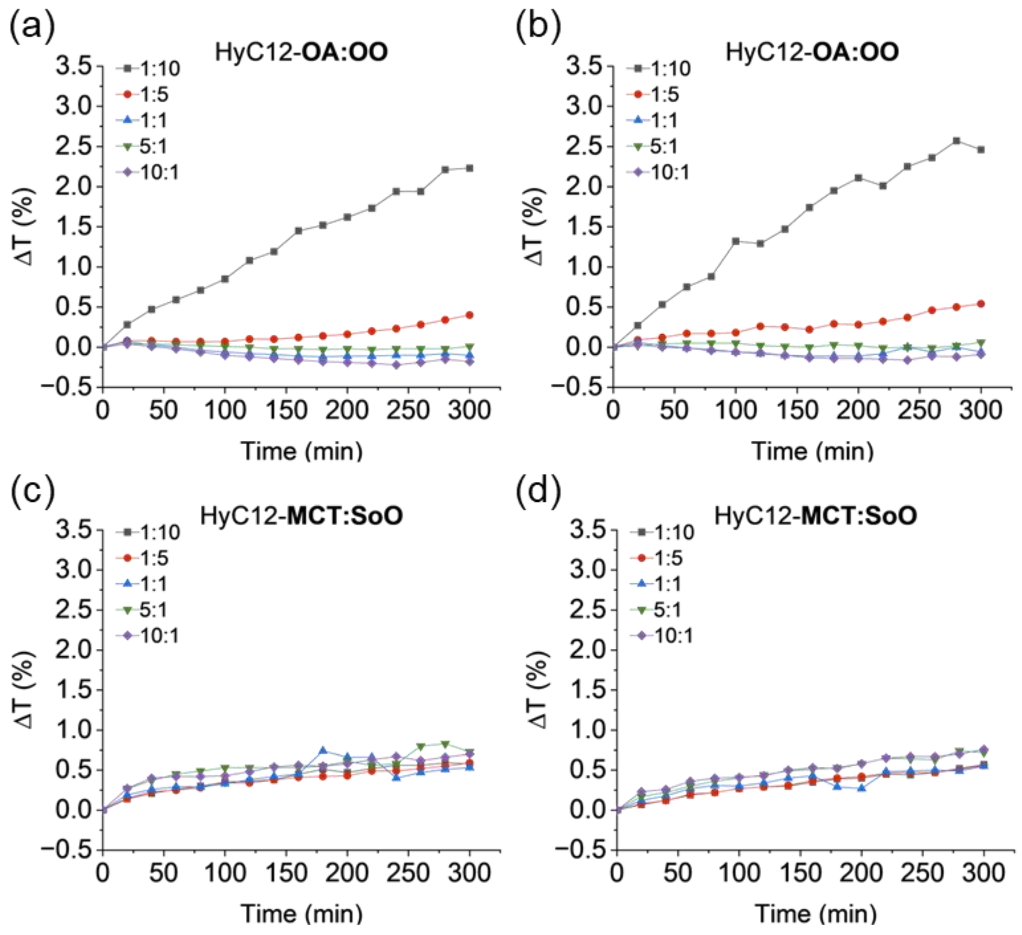

Figure S6. Mean variations in transmittance in the middle (**a**, **c**) and upper (**b**, **d**) parts of the capsule samples with mixtures of oils of different viscosities.

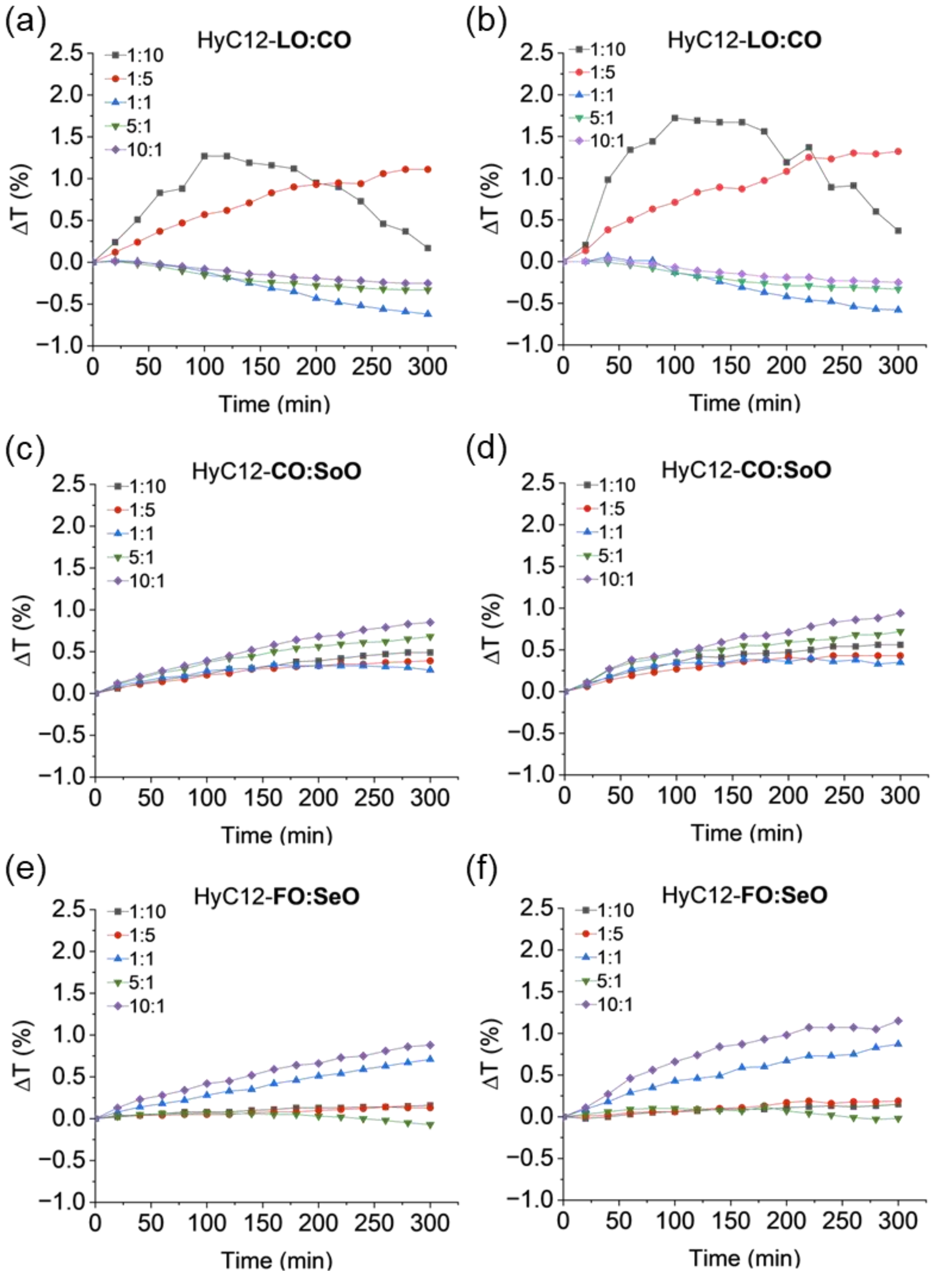

Figure S7. Mean variations in transmittance in the middle (**a**, **c**, **e**) and upper (**b**, **d**, **f**) parts of the capsule samples with different interfacial tension between aqueous and oil phases.

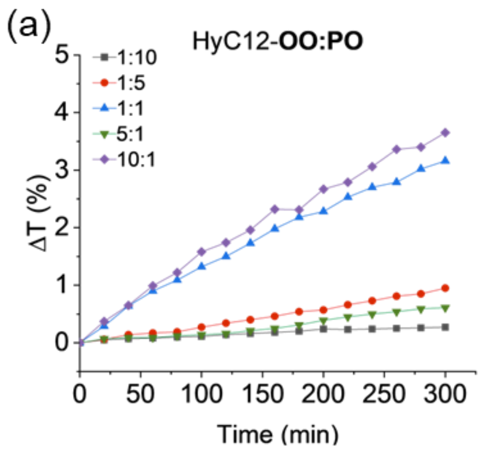

Figure S8. Mean variations in transmittance in the middle (**a**) and upper (**b**) parts of the capsule samples with mixtures of oils with similar physical properties.
